# Supplementary material for: 3,4-Methylenedioxypyrovalerone (MDPV) Sensing Based on Electropolymerized Molecularly Imprinted Polymers on Silver Nanoparticles and Carboxylated Multi-Walled Carbon Nanotubes
Source: Nanomaterials (Basel). 2021 Feb 1;11(2):353. doi: 10.3390/nano11020353 (PMC7912732; doi:10.3390/nano11020353)
Supplement: Supplementary file 1 [file nanomaterials-11-00353-s001.pdf]

nanomaterials

Supplementary Information for

3,4-methylenedioxypyrovalerone (MDPV) sensing based on electropolymerized molecularly imprinted polymers on silver nanoparticles and carboxylated multi-walled carbon nanotubes

Rosa A.S. Couto, Constantino Coelho, Bassim Mounssef Jr., Sara F. de A. Morais, Camila D. Lima, Wallans T.P. dos Santos, Félix Carvalho, Cecília M.P. Rodrigues, Ataualpa A.C. Braga, Luís Moreira Gonçalves, and M. Beatriz Quinaz

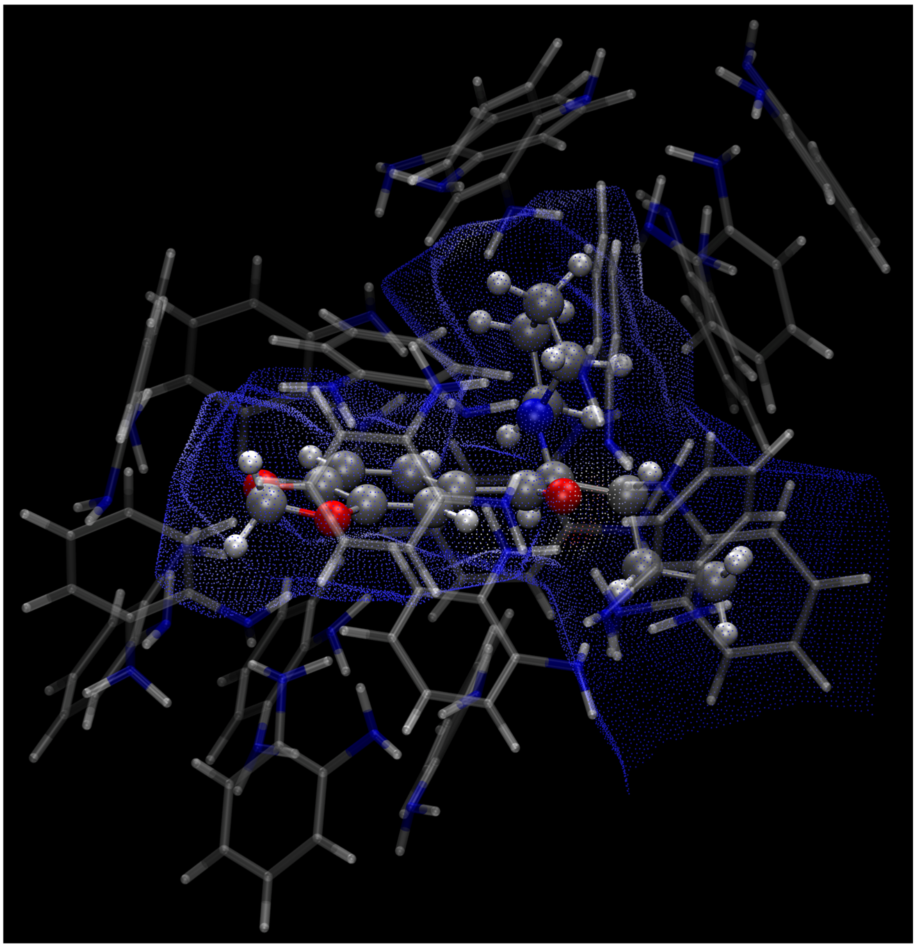

Figure S1. Becke surface of interactions between MDPV and *o*-PD monomers at the MIP pre-polymerization complex with 19 monomers.

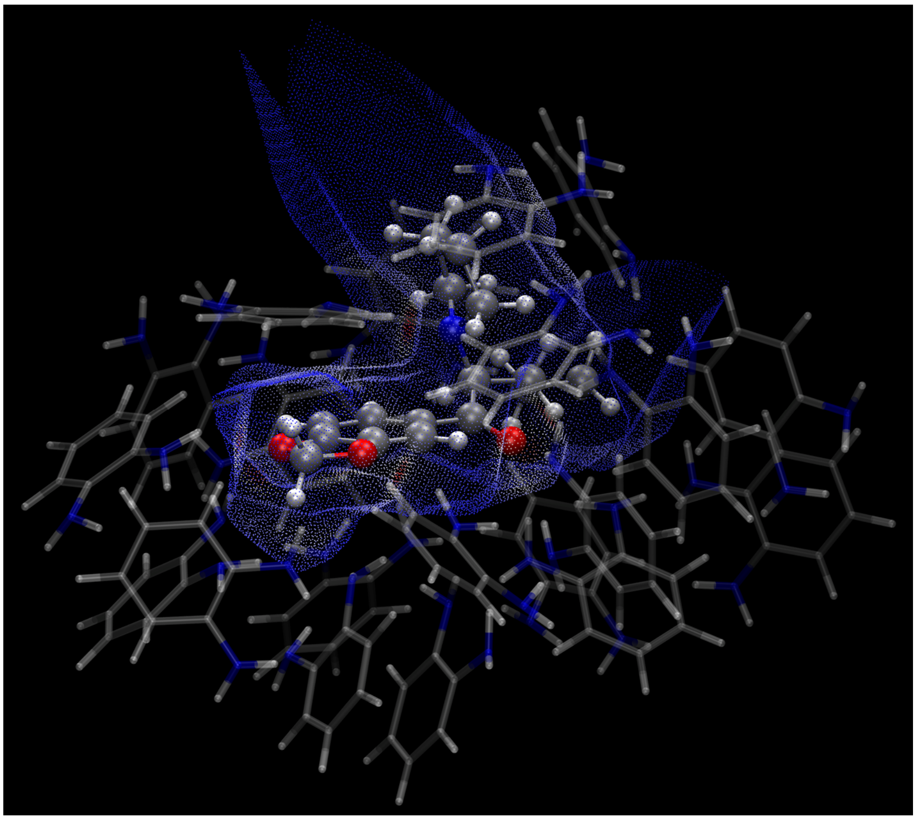

Figure S2. Becke surface of interactions between MDPV and *o*-PD monomers at the MIP pre-polymerization complex with 20 monomers.

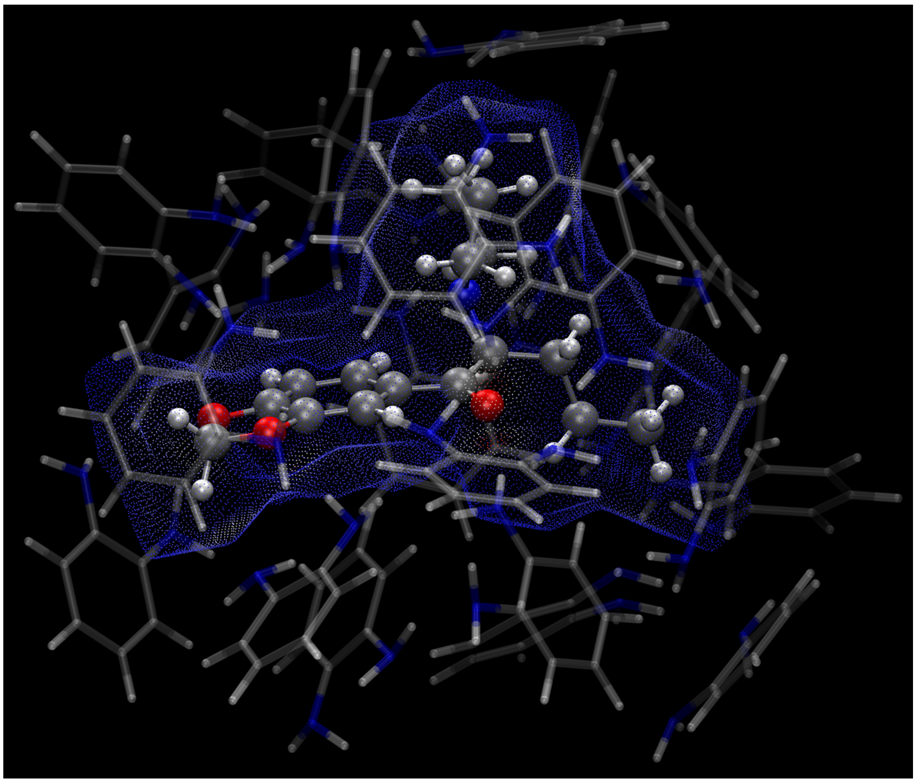

Figure S3. Becke surface of interactions between MDPV and *o*-PD monomers at the MIP pre-polymerization complex with 21 monomers.

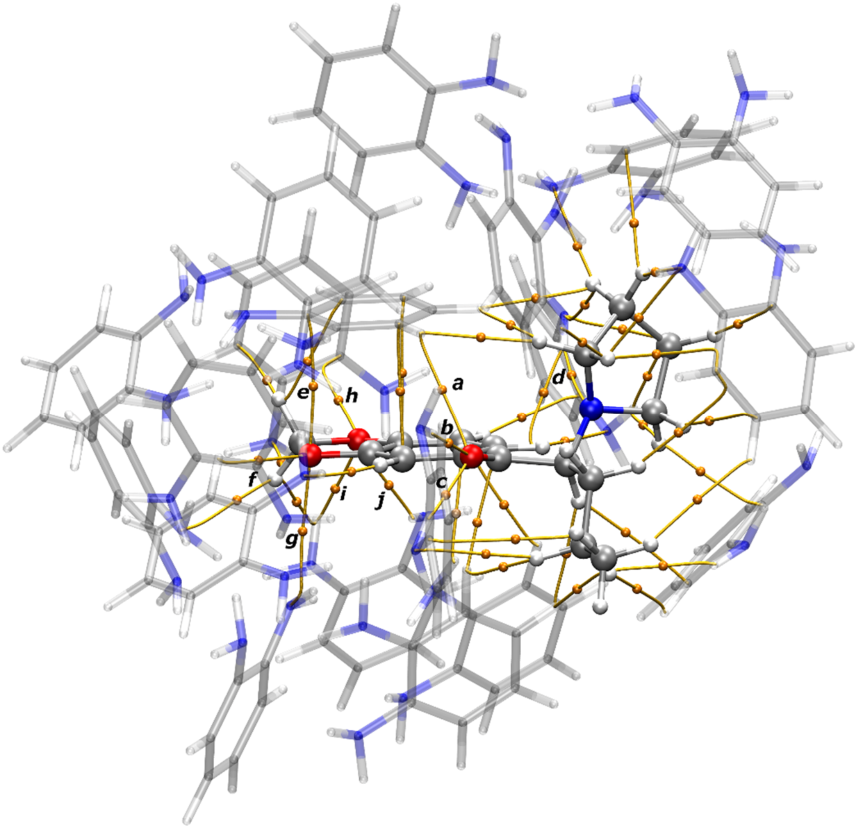

Figure S4. AIM molecular graph of the MIP pre-polymerization cavity with MDPV and 19 *o*-PD monomers. The orange lines are the Bond Paths (BP) that connect two attractors, and the orange spheres are the Bond Critical Points (BCP).

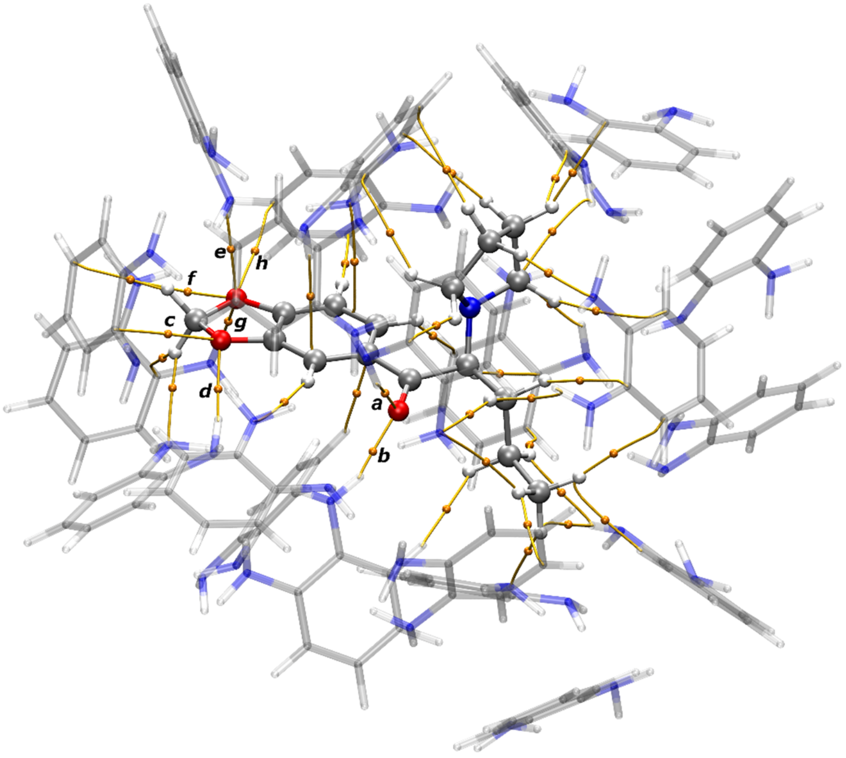

Figure S5. AIM molecular graph of the MIP pre-polymerization cavity with MDPV and 21 *o*-PD monomers. The orange lines are the Bond Paths (BP) that connect two attractors, and the orange spheres are the Bond Critical Points (BCP).

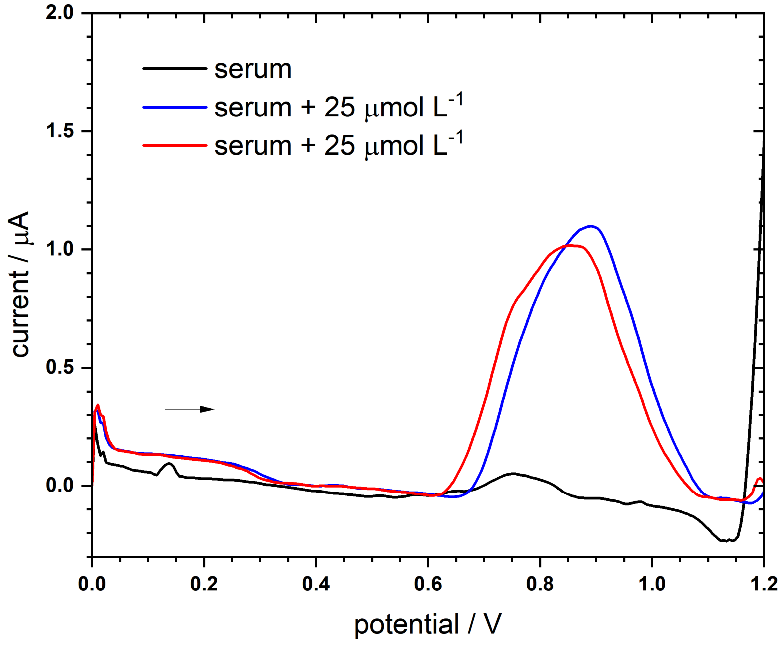

Figure S6. Blood serum (collected from healthy adults) diluted in PBS (1:20), blank and two different samples spiked with 25 μmol L^{-1}.

Table S1. AIM properties for the MIP pre-polymerization cavity with MDPV and 19 *o*-PD monomers and its respective Binding Energy (BE) calculated through the Equation of Espinosa. Electronic density ϱ(r), Laplacian of electronic density ∇^2ϱ(r), ellipticity ε, density of potential energy *V*(r) and density of total energy *H*(r), all data in atomic units and BE is in kcal mol^{-1}.

BCP	ϱ(r)	∇^2ϱ(r)	ε	*V*(r)	*H*(r)	*BE*
a	0.0149	0.0416	0.0657	-0.0105	-0.0001	-3.31
b	0.0057	0.0219	0.0696	-0.0036	0.0009	-1.13
c	0.0080	0.0256	0.0610	-0.0055	0.0004	-1.74
d	0.0080	0.0251	0.1608	-0.0054	0.0004	-1.70
e	0.0126	0.0365	0.0396	-0.0089	0.0001	-2.79
f	0.0159	0.0341	0.0711	-0.0094	-0.0005	-2.96
g	0.0072	0.0243	0.0693	-0.0048	0.0006	-1.52
h	0.0151	0.0466	0.0866	-0.0113	0.0002	-3.55
i	0.0059	0.0248	0.9471	-0.0042	0.0010	-1.31
j	0.0071	0.0251	0.0847	-0.0048	0.0008	-1.49
k	0.0046	0.0159	33.1546	-0.0028	0.0006	-0.89
l	0.0144	0.0453	0.1360	-0.0108	0.0003	-3.38
m	0.0140	0.0405	0.0557	-0.0103	-0.0001	-3.24

Table S2. AIM properties for the MIP pre-polymerization cavity with MDVP and 20 *o*-PD monomers and its respective Binding Energy (BE) calculated through the Equation of Espinosa. Electronic density ϱ(r), Laplacian of electronic density ∇^2ϱ(r), ellipticity ε, density of potential energy *V*(r) and density of total energy *H*(r), all data in atomic units and BE is in kcal mol^{-1}.

BCP	P	∇^2ϱ	ε	*V*(r)	*H*(r)	*BE*
a	0.0034	0.0142	1.4690	-0.0019	0.0008	-0.59
b	0.0200	0.0685	0.0355	-0.0143	0.0014	-4.49
c	0.0251	0.0838	0.0504	-0.0178	0.0016	-5.60
d	0.0043	0.0137	1.0804	-0.0023	0.0006	-0.72
e	0.0073	0.0231	0.1224	-0.0044	0.0007	-1.37
f	0.0034	0.0145	0.1970	-0.0019	0.0009	-0.59
g	0.0034	0.0162	2.2320	-0.0022	0.0009	-0.70
h	0.0069	0.0244	3.3283	-0.0041	0.0010	-1.28
i	0.0069	0.0263	0.0607	-0.0051	0.0007	-1.60
j	0.0087	0.0307	0.1780	-0.0065	0.0006	-2.04

Table S3. AIM properties for the MIP pre-polymerization cavity with MDVP and 21 *o*-PD monomers and its respective Binding Energy (BE) calculated through the Equation of Espinosa. Electronic density ϱ(r), Laplacian of electronic density ∇^2ϱ(r), ellipticity ε, density of potential energy *V*(r) and density of total energy *H*(r), all data in atomic units and BE is in kcal mol^{-1}.

BCP	ϱ	∇^2ϱ	ε	*V*(r)	*H*(r)	*BE*
a	0.0257	0.0837	0.0417	-0.0185	0.0012	-5.82
b	0.0253	0.0867	0.0303	-0.0181	0.0018	-5.67
c	0.0060	0.0194	0.4602	-0.0034	0.0007	-1.06
d	0.0181	0.0509	0.0410	-0.0129	-0.0001	-4.06
e	0.0119	0.0353	0.0308	-0.0090	-0.0001	-2.84
f	0.0066	0.0218	0.2523	-0.0043	0.0006	-1.35
g	0.0173	0.0483	0.0593	-0.0127	-0.0003	-4.00
h	0.0055	0.0191	0.1677	-0.0030	0.0009	-0.94
